# English Grammar Skills in Dutch Grade 4 Children: Examining the Relation Between L1 and L2 Language Skills

**DOI:** 10.1007/s10936-023-09968-x

**Published:** 2023-05-15

**Authors:** Margreet van Koert, Nihayra Leona, Judith Rispens, Jurgen Tijms, Maurits van der Molen, Hernán Labbé Grunberg, Patrick Snellings

**Affiliations:** University of Amsterdam, Amsterdam, The Netherlands

**Keywords:** Second language learning, Linguistic transfer, Relation between grammar and vocabulary

## Abstract

Second language proficiency may be related to first language acquisition (Ganschow & Sparks, 1991), but relatively little is known about the relation between first and second language grammatical proficiency in primary school children who are in their first stages of foreign language learning. This study aims to determine whether differences in Dutch and English vocabulary and Dutch grammar skills predict differences in English grammatical proficiency in Dutch speaking children who are in grade 4 in primary school. The selected participants are monolingual Dutch pupils (*N* = 152), aged 9;0–10;0. To measure the children’s vocabulary the PPVT was used in Dutch (Schlichting, 2005) and in English (Dunn & Dunn, 2007). In addition, two grammar tasks in English and one in Dutch of the CELF (Semel et al., 2003) were used. The results show that English vocabulary is a strong predictor of English grammar skills, and that the Dutch vocabulary skills are weaker predictors of English grammar skills. Moreover, Dutch grammar skills predict English grammar skills for one of the grammar tasks. These results are discussed vis-à-vis hypotheses about cross-domain transfer and cross-linguistic transfer (Blom et al., 2012; Cummins, 1979; Ganschow & Sparks, 1991; Paradis, 2011; Sparks, 1995).

## Introduction

Accounting for individual differences in language learning has been crucial to the field of second language acquisition (SLA). It is well established that there is a continuum of language learning, which spans from very poor second language learners to nativelike foreign language users (Hyltenstam & Abrahamsson, 2012; Sparks, 1995). Whereas many studies have addressed the issue of individual differences for adolescent or adult learners in an educational setting or for child learners in a naturalistic environment (e.g., in the speech community), it is not yet understood which factors best predict individual differences in the early stages of foreign language learning (both grammar and vocabulary) for young learners in an educational setting. In addition, relatively little is known about the relationship between first and foreign language proficiency in primary school children who are in the early stages of foreign language learning. The present study aims to determine whether there is a cross-linguistic relationship in the grammatical and vocabulary domain during those early stages.

### Relation Between L1 and Fl/L2^1^

Many factors play a role in foreign language learning. Language aptitude is one of the cognitive variables that has received its fair share of attention in SLA studies, especially when it comes to the relationship between native language (L1) skills and foreign language (FL) learning (Abrahamsson & Hyltenstam, 2008; Courtney et al., 2017; Ganschow & Sparks 1991; Geva, 2016; Li, 2016; Paradis, 2011; Sparks, 1995; Sparks et al., 2006; Sparks et al., 2008; Sparks et al. 2009a; Sparks et al. 2009b; Sparks et al., 2012). Language aptitude tests often include several components measuring different aspects of language learning ability, such as sensitivity to sound/symbol relationships, sensitivity to linguistic structure, and memory for vocabulary (Sparks et al., 2009a; Zurawsky, 2006). L1 proficiency is also a factor that is considered to be of importance. For instance, L1 reading skills, which are tested in primary school, correlate with students’ FL reading abilities in high school (Cummins, 1979; Ganschow & Sparks, 1991; van de Ven et al., 2018). Based on this type of evidence, several hypotheses have been put forward that reflect the idea that L1 skills form the basis for learning an FL (Cummins, 1979; Ganschow & Sparks, 1991; Koda, 2005). The linguistic coding differences hypothesis further suggests that if a learner has problems in their L1 with one aspect of language, for example phonological processing, then this may well have repercussions for their word decoding skills, vocabulary acquisition and even syntactic skills, in their L1 and in their FL (Sparks, 1995).

The relation between one language and the other is also apparent in cross-linguistic transfer. Studies (cf., Blom et al., 2012; Paradis, 2005; Paradis, 2011; Paradis et al., 2008) that investigated the (oral) production of verbal morphology of L2 English in children with typologically different L1s showed that little of the L1 is transferred during the early stages of English L2 acquisition in a naturalistic setting. In these studies English was the official language of the speech community. No differences were found between children whose L1 contained inflection, such as Spanish, and children whose L1 was an isolating language, i.e. had little to no inflection, such as Chinese (Paradis, 2005; Paradis et al., 2008). However, when children were tracked over a longer period of time (Blom et al., 2012), or when they were tested after a longer period of L2 exposure (Paradis, 2011), their L1 became a significant predictor of their verbal morphology production, showing an effect of L1 transfer. The grammatical properties that are present in their L1, such as tense or agreement markers, can be transferred to the L2 if those same properties are present in the L2 too.

A widely held assumption is that these grammatical properties are learned through learning mechanisms that also underlie vocabulary acquisition (Blom et al., 2012; Kohnert et al., 2010; Paradis 2011), which is a feature of the usage-based framework in which lexicon and grammar are not considered as distinct and separate mechanisms (Bybee, 2010). The cross-domain relationship within a language between vocabulary and grammar has been found when examining word frequency (Blom et al., 2012) and lexicon size (Kohnert et al., 2010). L2 verbs with higher frequency tend to be more accurately inflected by young L2 learners (Blom et al., 2012) and young L2 learners with greater vocabulary knowledge typically produce longer utterances in their L2 (Kohnert et al., 2010), thereby displaying greater grammatical ability. Those young L2 learners show the same pattern in their L1, which means that learners with a greater L1 vocabulary knowledge also utter longer L1 sentences (Kohnert et al., 2010). All in all, the results of these studies show that there are cross-linguistic relationships between the L1 and the L2, and cross-domain relationships between the vocabulary and grammar during early language acquisition in naturalistic settings.

### L1 Development: Vocabulary and Grammar

During the early stages of L1 development, vocabulary growth and grammatical development are intertwined. A study by Bates and Goodman (1997) found a strong correlation between vocabulary growth, between 8 and 30 months of age, and grammatical complexity, between 16 and 30 months of age, on the basis of parental report. Kidd and Kirjavainen (2011) also found a strong correlation between grammar and the lexicon. They examined the production of verbal inflectional morphology by Finnish children, aged between 4;0 and 6;7, and found a positive relation between the size of the lexicon and verbal morphology production. Together the two variables predicted the children’s performance on all the different verb types. Hence, both studies found that lexical knowledge and grammatical knowledge strongly correlate. During the initial stages of language development, a critical mass of vocabulary knowledge seems necessary for the growth of grammar (Bates & Goodman, 1997). This does not mean that vocabulary is required for all types of grammatical structures (Brinchmann et al., 2018), as it has been shown that infants already master abstract grammatical constructions, such as word order (Benavides-Varela & Gervain, 2017; Gervain & Werker, 2013).

One of the explanations for the tight relationship between vocabulary and grammar is given by Elman (1990, 1993) and Bybee (2008). As briefly stated above, usage-based grammar (Bybee, 2008, 2010) hypothesizes that experience with language and frequency of linguistic items in the input shape the cognitive organisation of language, leading to a network where information about form and meaning is intertwined and stored together. As language experience of a person drives the organisation and abstraction of linguistic features of perceived linguistic input, it is not surprising that frequency and language input is central to language development in this framework. Vocabulary is important for developing grammar skills as lexical items contain grammatical markers (for instance ‘ed’ representing here the regular past tense: play-ed; walk-ed; comb-ed). Grammatical information in such lexical items is only extracted after a certain amount of lexical items with similar grammatical affixes have been encountered. In addition, this hypothesis could explain why children who know more vocabulary items, as in Kidd and Kirjavainen’s study (2011), also have access to more grammatical derivations.

### Fl/L2 Development: Vocabulary and Grammar

What about cross-domain relationships in FL? Some studies give evidence for a tight relationship between lexical and grammatical skills in an FL. Martin and Ellis (2012) investigated university students who learned an artificial language with intricate morphological rules, without being told those rules (implicit learning). They found a robust relationship between vocabulary and grammar scores with intercorrelations of 0.44 to 0.76, suggesting a moderate to strong interdependence. This finding strongly suggests that vocabulary and grammar were related for these adults learning a completely new language. As mentioned above, the study by Kohnert et al. (2010) investigated the link between words and grammar in sequential bilingual pre-schoolers, who learned their L2 (English) through the speech community. They reported strong correlations between words and grammar in the L2 of the participants (English). Service and Kohonen (1995) examined Finnish primary school children aged 12;3 on their knowledge of English, including listening, reading, essay writing and vocabulary, and on their phonological short-term memory capacity. The results showed that FL English vocabulary accounted for much of the variance of the English language test in the fixed order regression analysis after the mean of all their grades – serving as a proxy for IQ – had been entered. These findings demonstrated that vocabulary and grammar were strongly related for these young learners who had received English training in education for two or three years. However, although grammar was included in this study, it was part of comprehension tests, which measured many aspects of language besides grammar, and of cloze tests, which rely heavily on vocabulary knowledge. Therefore, it is not straightforward to tease apart the contribution of grammatical knowledge from lexical knowledge in this study. The correlations between vocabulary and grammar fit the Usage-based account of language learning as it does not have qualitatively different predictions for FL than L1, under the assumption that there must be a fair amount of input from the FL (Bybee, 2008).

### English in the Netherlands

English holds a special position in the Netherlands. First, English and Dutch are linguistically closely related. Typological proximity plays a role in foreign language acquisition (cf., Lindgren & Muňoz, 2013). Second, English is omnipresent in the Netherlands, as many TV programmes are in English with Dutch subtitles and as many songs on the radio are in English. Therefore, English is not a typical FL in the Netherlands because people are too familiar with English for it to be an FL; however, it is also not a second language, as English is not an official language of the Netherlands. Because of this reason we refer to English as an FL in the current study. Unlike other FLs that are taught at secondary school (for example German or French), English education starts in primary school (Toorenburg & van Oostdam, 2002). Many studies have investigated English language learning at primary and secondary schools (e.g., Driessen et al., 2016; Goriot et al., 2018a; Goriot et al. 2018b; de Graaff, 2015; Toorenburg & van Oostdam, 2002; Unsworth et al., 2015). However, to our knowledge none of those studies explicitly linked Dutch vocabulary and grammatical knowledge to the first stages of English vocabulary and grammar learning in an educational setting.

### The Current Study

Collectively, the data available to date indicate that there is a relationship between L1 and FL proficiency. Furthermore, it has been shown that vocabulary and grammar are related in the early stages of L1 acquisition. In addition, evidence suggests that FL vocabulary and FL grammar may be related. Nevertheless, a number of important questions need to be addressed. First of all, are L1 vocabulary and grammar related in children who have outgrown the early stage of grammatical development? There is surprisingly little evidence in the literature about the linguistic and in particular the grammatical development in children at the end of primary school. For monolinguals it has been found that the strength of word-grammar relationships declines over time (Kohnert et al., 2010). It could be that children until kindergarten age 4 rely on their vocabulary to advance their grammatical knowledge but that children around the ages of 9–10 do not need this reliance on vocabulary anymore, because their L1 grammar is already in place. Nevertheless, at present this is mere speculation and we therefore address the association between vocabulary and grammar in L1 in primary school children in this study.

Secondly, are FL vocabulary and FL grammar related in young, beginning learners in an educational setting? Despite extensive research into the learning of FL reading skills in of older age groups (e.g., Bernhardt & Kamil 1995; Van Gelderen et al., 2004; Droop & Verhoeven, 2003), the learning of FL grammar in this young group of learners appears under researched. Martin and Ellis (2012) demonstrated that adults, learning an artificial language, show a strong correlation between vocabulary and grammar; however, it is not clear whether young, beginning FL learners exhibit the same correlation. An indication of this association would confirm the hypothesis of the usage-based framework.

Finally, is it the case that children who have a better understanding of grammar in their L1 are the same children who have a better grasp of their FL grammar? Previous evidence suggests that L1 skills lay the foundation for FL learning (Cummins, 1979; Ganschow & Sparks, 1991), but is it unclear whether this holds for grammar skills. In the present study, grammar is operationalized as FL oral comprehension and FL morpho-syntax. To the best of our knowledge, no studies have directly compared L1 and FL grammar in children learning an FL in an educational setting. The current study aims to address this issue.

## Method

### Participants

Two hundred and ninety-eight grade 4 pupils across seven primary schools in the Randstad, the conurbation in the western part of the Netherlands, took part in the first round of data collection. Parental permission (active consent) was obtained for each participant. The study was approved by the Ethical Review Board of the university. The schools had different pupil populations regarding social-economic status based on their postal codes and regarding the number of children with Dutch as an additional language based on the answers the participants gave in the language background questionnaires. Two schools were situated in an urban area, four in a suburban area and one in a rural area. According to national Dutch normative data, the schools were situated in neighbourhoods from low to middle social-economic status (SCP, 2016). Hence, although our sample was a convenience sample, we took care to recruit schools serving students of different economic status and from both urban and rural areas. Four of the seven schools offered English language lessons before grade 4, but no differences on the crucial measures were found between the participants who had received English language lessons and those who had not.^2^ In the present study the data from dyslexic children (*N* = 16) were excluded, as were the data from children who had a mother tongue other than Dutch (*N* = 123). The reason for these exclusions was that we carefully wanted to examine the relation between Dutch and English, without interference from other languages or language impairments. Other than obtaining on average lower scores on the Dutch tasks, these excluded children did not behave very differently as a group from the participants in the present sample. Finally, some participants did not complete all of the relevant tasks and those data were excluded, too (*N* = 7). Hence, the total number of participants was 152. There were 76 male and 76 female participants. The mean age of the participants was 9 years and 10 months. The participants were tested half-way through the school year.

### Instruments

#### Raven

The measure of the non-verbal intelligence was the RAVEN Standard Progressive Matrices (RAVEN-NL SPM, Dutch adaptation by Harcourt Assessment, 2006). The RAVEN consists of five blocks, each containing 12 items (*N*_RAVEN_ = 60). Each item consists of a geometric pattern with one piece missing. Participants have to discover the principles, relationships and rules between the patterns and decide which of six given pieces is the missing one. A test-retest reliability of 0.88 was reported for the test (Raven, 2006). This task was used to measure participants’ logical reasoning skills and to ascertain whether participants with better logical reasoning skills would also have higher grammatical sensitivity, as this is typically found (Skehan, 1998; Wesche, 1981; but see Sternberg, 2002 for a different interpretation of these results). The present administration differed slightly from standard administration in two ways. First of all, participants took the test on a tablet (see Procedure for more information) instead of with pencil and paper. Secondly, participants had 30 min to complete the test (see Hamel & Schmittman, 2006 for validation of this procedure). Finally, participants received one point for each correct answer, leading to a score out of 60.

#### L1 Vocabulary

The measure of L1 vocabulary was the Peabody Picture Vocabulary Test, Third edition (PPVT-III-NL, Dunn & Dunn, 2007; Dutch translation by Schlichting 2005). The test provides an estimate of receptive vocabulary for standard Dutch. The present administration differed from standard administration in two ways. First of all, participants took the test on a tablet (see Procedure for more information) rather than using a booklet. Participants saw four yellow and blue coloured pictures and heard a sound file containing the target word that was played automatically. The sound file was recorded by a female native speaker of Dutch. Participants had to select the correct picture, out of four pictures, upon hearing the target word. If they did not respond within five seconds, they received a warning message saying *select a picture*. Secondly, since this test was administered in class, all of the participants completed sets 7 up and including 11 (*N*_PPVT−NL_ = 59), regardless of how many mistakes they made in a set.^3^ These sets were selected, as they were deemed appropriate for this age group. Hence, no basal or ceiling rules were used. Finally, participants received one point for each correct answer, leading to a score out of 59.

#### L1 Sentence Assembly

L1 sentence production was examined by using the task Sentence Assembly from the Clinical Evaluation of Language Fundamentals (CELF-4-NL, Semel et al., 2003; Dutch adaptation by Kort et al., 2010). There were two trials and 13 exercises (*N*_CELF−ZS_ = 13). This test was administered to assess the participants’ ability to formulate grammatically acceptable and semantically meaningful sentences by manipulating and transforming given words and word groups. Almost all of the words in the sentence assembly task were high frequent, basic words. 4 An example of a test item is given in (1) and examples of possible grammatically acceptable and semantically meaningful sentences of the word groups in (1) are given in (2) and (3).

1. [weet] [niet] [hij] [hij het] [of] [verstuurd heeft].
  [knows] [not] [he] [he it] [if] [sent has]

(2) [hij] [weet] [niet] [of] [hij het][verstuurd heeft].
  [he] [knows] [not] [if] [he it] [sent has]
  ‘He does not know whether he sent it.’

(3) [weet] [hij] [niet] [of] [hij het] [verstuurd heeft].
  [knows] [he] [not] [if] [he it] [sent has]
  ‘Does he not know whether he sent it?’

This task was used to assess L1 grammar knowledge in previous studies (Justice et al., 2010; Rescorla, 2002; Weber-Fox & Neville, 1996). The present administration differed slightly from standard administration in two ways. First of all, participants took the test on a tablet (see Procedure for more information) instead of using a booklet. Participants saw word groups on the screen of their tablet and had to type in two grammatically correct sentences for each item. If both sentences were correct, the participant received one point for that item. Secondly, since the CELF-manual allowed very few sentences to count as correct, we listed all the grammatically correct and semantically meaningful versions of the sentences and manually judged those to be correct. Typos, punctuation and spelling errors were ignored.

#### FL Vocabulary

The measure of FL vocabulary was the Peabody Picture Vocabulary Test, Fourth edition (PPVT-4, Dunn & Dunn 2007).^5^ The test measures receptive vocabulary knowledge. The present administration differed from standard administration in two ways. First of all, participants took the test on a tablet (see Procedure for more information) instead of a booklet. Participants were presented with four coloured pictures and heard a sound file containing the target word that was played automatically. The sound file was recorded by a female native speaker of British English. Participants had to select the correct picture, out of four pictures, upon hearing the target word. If they did not respond within five seconds, they received a warning message saying *select a picture*. The second difference from standard administration was that all of the participants completed sets 1 up and including 7 (*N*_PPVT−EN_ = 61), regardless of how many mistakes they made in a set, because the test was administered in class.^6^ These sets were selected, as they were deemed appropriate for this age group. Hence, no basal or ceiling rules were used. Finally, participants received one point for each correct answer, leading to a score out of 61.

#### FL Oral Comprehension

FL oral comprehension was examined by using the task Sentence Structure from the Clinical Evaluation of Language Fundamentals (CELF-4, Semel et al., 2003). There were three trials and 26 test sentences (*N*_CELF−SS_ = 26). The test was administered to assess the participants’ ability to interpret spoken sentences and to select the pictures that illustrate referential meaning of the sentences. This test was used to assess L1 grammatical knowledge in previous studies (Justice et al., 2010; Rescorla, 2002; Weber-Fox & Neville, 1996). The present administration differed slightly from standard administration. Participants had to perform on a tablet (see Procedure for more information) instead of using a booklet. Participants were presented with four coloured pictures and heard a sound file containing the target sentence that was played automatically. The sound file was recorded by a female native speaker of British English. Participants had to select the correct picture, out of four pictures, upon hearing the target sentence. Finally, participants received one point for each correct answer, leading to a score out of 26.

#### FL Morpho-Syntax

Word Structure from the Clinical Evaluation of Language Fundamentals (CELF-4, Semel et al., 2003) was used to examine FL morpho-syntactic production. It was administered to measure the participants’ ability to apply morphological rules to mark inflection and comparison and their ability to select and use appropriate pronouns to refer to people. This test was used to examine L1 grammatical knowledge in previous studies (Justice et al., 2010; Rescorla, 2002; Weber-Fox & Neville, 1996). There were 32 items in the original test, but we chose to simplify it, on the basis of a pilot study, by reducing the number of items to 12 (*N*_CELF−WS_ = 12). The present administration differed slightly from standard administration. Participants were presented with a coloured picture and a sound file containing the target sentence. The sound file was recorded by a female native speaker of British English. Participants were asked to complete the target sentence in English. All of the instructions were in Dutch. Finally, participants received one point for each correct answer, leading to a score out of 12.

### Procedure

All of the tests were part of a larger test battery in a three-year longitudinal study into the predictors of English language learning by Dutch primary school pupils. Testing began in February for four schools, in March for two schools and in April for one school and lasted about three months, depending on the availability of the school. The test battery consisted of class sessions, in which participants worked by themselves in their classroom, and individual sessions, in which a test administrator tested a participant individually in a separate room. In the larger test battery, there were five class sessions and six individual sessions. Participants carried out the tests by themselves on a tablet in the class sessions; they wore headphones and a short video served as an instruction for each test. Three of the class sessions contained Dutch (L1) language tests and the other two included English (FL) language tests. Each participant had a tablet (T550 Galaxy Tab A 9.7) to work on. The Dutch (L1) and English (FL) vocabulary tests, the Dutch (L1) sentence assembly and the English (FL) oral comprehension test were part of the class sessions. All the class tests were converted to an online survey (www.qualtrics.com), so that the participants’ answers were recorded and stored automatically and digitally. Each class session took about 45–60 min to complete. The language background and the English (FL) morpho-syntax tests were administered individually. Individual sessions took about 30 min to complete.

## Results

The mean scores of the 152 participants on the Raven and the Dutch (L1) and English (FL) measures are reported in Table 1. The IBM SPSS Statistics (Version 24, released 2016) was used for the statistical analyses of the reliabilities and of the correlations. The Raven scores fell in the normal range. The mean scores on the L1 and FL vocabulary tests were quite similar; however, the L1 items were suitable for native speakers between the ages of 6;6 and 15;11, whereas the FL items were suitable for native speakers between the ages of 2;6 and 8;0. Hence, the L1 vocabulary items were more difficult than the FL items. On average the participants scored around two-thirds correct on all the tests, except for the FL morpho-syntax task where they scored one-third correct. The internal consistency reliability of all the tasks was good, see Table 1. All of the measures were normally distributed.

**Table 1:** The mean scores, standard deviations, reliability, range of scores and number of items per test

	*M*	*SD*	α	Range		No. of items
Raven	38.2	6.9	0.847	21–53	60	
L1 vocabulary	39.9	6.0	0.758	13–51	59	
L1 sentence assembly	8.6	2.6	0.720	0–13	13	
FL vocabulary	37.0	7.2	0.817	15–56	61	
FL oral comprehension	16.2	4.4	0.758	5–26	26	
FL morpho-syntax	3.7	2.9	0.790	0–11	12	

*Note*. *N* = 152

First we wanted to know whether L1 vocabulary and L1 grammar were related, as our first research question asked: are L1 vocabulary and grammar related in children who have outgrown the early stage of grammatical development? To determine whether this relationship held, we carried out a Pearson *r* correlation analysis. Table 2 reports the Pearson *r* correlation values for all the L1 and FL measures.

**Table 2:** Correlation matrix of the intelligence, L1 and FL lexical and grammatical measures

	Raven	L1 vocabulary	L1 sentence assembly	FL vocabulary	FL oral comprehension		FL morpho-syntax
Raven	1						
L1 vocabulary	0.26^**^	1					
L1 sentence assembly	0.18^*^	0.33^**^	1				
FL vocabulary	0.15	0.21^*^	0.07	1			
FL oral comprehension	0.18^*^	0.23^**^	0.05	0.64^**^		1	
FL morpho-syntax	0.12	0.28^**^	0.10	0.65^**^		0.68^**^	1

*Note. N* = 152, ^*^p < .05, ^**^p < .01

Table 2 shows that there was a moderate positive correlation between L1 vocabulary and L1 sentence assembly (*r* = .33, *p* < .001), meaning that participants who had a larger vocabulary in Dutch, also performed better on L1 sentence assembly, and vice versa.

We also wanted to know whether FL vocabulary and FL grammar were related. Our second research question asked: Are FL vocabulary and grammar related in young, beginning learners in an educational setting? Table 2 shows that there was a strong positive correlation between FL vocabulary and FL oral comprehension (*r* = .64, *p* < .001) and between FL vocabulary and FL morpho-syntax (*r* = .65, *p* < .001), indicating that participants who had a larger vocabulary in English, also performed better on the English grammar tasks, and vice versa.

Our third and final research question asked whether it is the case that children who have a better understanding of grammar in their L1 are the same children who have a better grasp of their FL grammar even when we take the predictive role of logical reasoning skills and FL vocabulary into account? The statistical analysis for this research question was done in R (Core Team, 2018) with generalized (logistic) linear mixed-effects models using the glmer function of the lme4 package (Bates et al., 2015). The *p* values were obtained with the lmerTest package (Kuznetsova et al., 2017). Models were built with all four predictors as fixed effects (Raven, L1 vocabulary, L1 grammar, FL vocabulary). The random structure was kept maximal (Barr et al., 2013) with random intercepts for subject and item, and random slopes for every predictor. Confidence intervals were calculated with the confint function using the Wald method.

Two models were built following these specifications. One for each dependent variable (FL oral comprehension and FL morpho-syntax). A summary of the results of both models can be seen in Table 3. Estimated effects are rounded to three decimals and represent odd-ratios.

**Table 3:** * A summary of the results of the models for FL oral comprehension and FL morpho-syntax*

Model	Predictor	Estimate	95% CI		*z*	*p*
			*LL*	*UL*		
FL oral comprehension	Raven	0.996	0.984	1.008	− 0.646	0.518
	L1 sentence assembly	1.002	0.994	1.009	0.409	0.683
	L1 vocabulary	1.017	1.002	1.032	2.185	0.029
	FL vocabulary	1.062	1.043	1.081	6.588	0.001
FL morpho-syntax	Raven	0.982	0.963	1.001	-1.877	0.060
	L1 sentence assembly	1.017	1.005	1.030	2.812	0.005
	L1 vocabulary	1.04	1.010	1.071	2.623	0.009
	FL vocabulary	1.12	1.094	1.147	9.324	0.001

The third research question asked whether L1 vocabulary, L1 grammatical knowledge, logical reasoning and FL vocabulary predicted FL grammatical knowledge. First we look at FL oral comprehension. The results of the model with FL oral comprehension as dependent variable show that only L1 vocabulary and FL vocabulary are significant predictors of the score participants obtained in the FL oral comprehension test. Specifically, L1 vocabulary scores increase the odd-ratio of scoring more correctly in the FL oral comprehension task by 1.017. This effect was statistically significant (*p* = .029, *z* = 2.185, with a 95% confidence interval from 1.002 to 1.032). FL vocabulary increases the odd-ratio of scoring more correctly in the FL oral comprehension test by 1.062. This difference is significant (*p* < .001, *z* = 6.588, with a 95% confidence interval from 1.043 to 1.081). Next, we look at FL morpho-syntax, as the third research question asked whether L1 vocabulary, L1 grammatical knowledge, logical reasoning and FL vocabulary was associated with FL grammatical knowledge. The results of the model with FL morpho-syntax as the dependent variable show that L1 grammatical knowledge, L1 vocabulary and FL vocabulary increase the odd-ratio of having more correct scores on the FL morpho-syntax task. Specifically, L1 grammatical knowledge increases the odd-ratio of scoring more correctly on the FL morpho-syntax task by 1.017. This effect is significant (*p* = .005, *z* = 2.812 with a 95% confidence interval from 1.005 to 1.03). L1 vocabulary increases the odd-ratio of obtaining more correct scores on the FL morpho-syntax task by 1.04. This effect is significant (*p* = .009, *z* = 2.623 with a 95% confidence interval from 1.01 to 1.071). Finally, the strongest predictor of the FL morpho-syntax task is FL vocabulary, which increases the odd-ratio of obtaining more correct scores in the FL morpho-syntax task by 1.12. This effect is significant (*p* < .001, *z* = 9.324 with a 95% confidence interval from 1.094 to 1.147).

## Discussion

The aim of the present study was to determine whether differences in Dutch vocabulary and grammar knowledge in Dutch speaking pupils predict differences in grammatical proficiency in English, whilst accounting for the English vocabulary level and logical reasoning skills, in the initial stages of educational language learning. Participants were Dutch primary school pupils who completed vocabulary and grammar tasks in Dutch and in English.

We expected that L1 lexical and L1 grammatical knowledge were positively related. The results showed a significant, albeit moderate, positive relationship was obtained, indicating that the 9-year-old Dutch pupils who knew more words on the Dutch vocabulary task were significantly more likely to obtain more correct scores on the Dutch sentence assembly task than their peers who knew fewer words. This result is in line with findings from prior research, which found an even stronger positive correlation between word and grammar knowledge in the L1 (Bates & Goodman, 1997; Brinchmann et al., 2018; Hoff et al., 2018; Kidd & Kirjavainen, 2011; Kohnert et al., 2010, but see Hoff et al., 2018 for a different interpretation) and is in line with the predictions of the usage-based framework (Bybee, 2008, 2010). These previous studies established this relationship for early L1 acquisition; however, the present study shows that in L1 development vocabulary and grammar are still related in children who are relatively advanced in their linguistic development. What makes this finding even more meaningful is that the L1 sentence assembly task only contained well-known, highly frequent words; hence, participants likely did not differ in their lexical skills needed to carry out this task and therefore differences in task performance can be attributed to differences in grammatical proficiency only. Participants had to rely on their syntactic knowledge to combine the word groups into grammatically correct and semantically meaningful sentences. The participants who scored low had difficulties forming two distinct grammatically correct and semantically meaningful sentences. They may have lacked richness in grammatical structures, meaning that the set of syntactic structures they had at their disposal was smaller than that of their peers. Our finding could indicate that a rich and extended vocabulary is related to cognitive representations of diverse grammatical structures.

A positive relationship was also expected between FL vocabulary and FL grammar. A strong correlation with vocabulary was observed for both the FL oral comprehension task, assessing receptive syntactic knowledge, and the FL morpho-syntactic task, assessing productive morphological knowledge. This finding suggests that the Dutch pupils who knew more words on the English vocabulary task were also significantly more likely to understand English sentences correctly and more likely to complete English sentences correctly than their peers who knew fewer words. This is in line with previous research (Goriot et al., 2018a; Kormos & Sáfár, 2008; Martin & Ellis, 2012; Kohnert et al., 2010; Service & Kohonen, 1995) and is in line with the usage-based grammar framework for second language acquisition. The current study is one of the few studies in which vocabulary was tested separately from grammar. Yet, there is a possible caveat. The grammar tasks used in the present study were intended for young monolingual English users and may well have imposed a considerable vocabulary demand. However, when we compared all the FL tasks, we observed only a small overlap, as only six words (*dog*, *duck*, *eating*, *flower*, *painting*, *happy*) appeared in the vocabulary task and in the FL oral comprehension task and/or in the FL morpho-syntactic task. Hence, the FL tasks differed in the words they employed. Moreover, the grammar tasks were designed to assess grammatical knowledge: the outcomes are a representation of how much syntactic knowledge of English these Dutch nine-year-old pupils have at their disposal. Although vocabulary was involved in the grammar tasks, they measured grammatical knowledge. Based on our findings, again the conclusion can be drawn that language experience is key. The more FL learners have been exposed to words, the more likely it is that they also have more experience with diverse grammatical structures (see for a similar account for young children Hoff et al., 2018).

Finally, it was hypothesised that FL grammar would be predicted by L1 vocabulary, L1 grammar and FL vocabulary. For the FL oral comprehension task it was found that both FL vocabulary and L1 vocabulary were significant predictors of the English oral comprehension task. This pattern indicates that the variation in the performance on the English oral comprehension task could, to a certain extent, be predicted by Dutch children’s English and Dutch vocabulary knowledge. A plausible explanation for this finding is that oral comprehension depends not only on grammatical comprehension but also on lexical comprehension. The choice of task may thus explain the absence of transfer of Dutch grammar to English oral comprehension. Kohnert et al. (2010, p. 695) express that studies that investigate transfer of specific morphosyntactic features may find specific instances of L1 transfer and they suggest that general measures of grammatical development may disguise specific instances of L1 transfer. In the present study it could be that the English oral comprehension task was too general a task (i.e., not diving into specific morphosyntactic features) to measure transfer of Dutch grammatical knowledge. Another explanation is that different types of grammatical knowledge were measured in the L1 and FL tasks. Some studies investigating early L2 acquisition did not obtain differences between children with an isolating L1 and an inflecting L1 (Paradis, 2005; Paradis et al., 2008). In these studies, the earliest stages of L2 acquisition were studied and Blom et al. (2012) suggest that low accuracy was one of the reasons for the absence of differences between isolating and inflecting L1s. Likewise, in the current study, it might be that the grammatical tasks used in the L1 and in the FL tapped into different types of syntactic knowledge. Whereas L1 sentence assembly relates to productive integrated knowledge of syntax morphology and semantics, the FL oral comprehension task relates to receptive syntactic knowledge.

The analyses of the FL morpho-syntax task painted a slightly different picture: both Dutch vocabulary and English vocabulary were again significant predictors, but Dutch grammatical knowledge also contributed to the scores on the English morpho-syntax task. Although it has been found that receptive vocabulary can be transferred from the L1 to the FL, children’s overall L1 vocabulary level does not seem to be predictive of FL vocabulary development (Goodrich et al., 2016). Why did Dutch vocabulary knowledge predict the FL morpho-syntactic task? As suggested above, it is likely that those children, who know more words, have more experience with language. Also, it has been reported that a larger vocabulary tends to be associated with a greater knowledge of syntax (Bates & Goodman, 1997) and of verbal morphology (Kidd & Kirjavainen, 2011). If the Dutch vocabulary task can be seen as a reflection of morphological skills (cf., Kidd & Kirjavainen 2011) that are not reflected yet in the English vocabulary measure, then this may explain why Dutch vocabulary knowledge predicts English morpho-syntactic skills. Why was Dutch grammatical knowledge one of the predictors of the English morpho-syntax task? Studies investigating children acquiring an L2 through the speech community have shown that L1 grammatical features can be transferred to the L2 if the L2 has the same features (Blom et al., 2012; Paradis, 2011). English and Dutch are closely related languages and have comparable tense and agreement markers potentially explaining that transfer of those properties was found in the present study.

What conclusion can be drawn from the present findings in light of transfer? Our study shows that this was task dependent: Dutch grammatical proficiency predicted the FL morpho-syntactic task. Dutch vocabulary, which may well be a reflection of morphological skills, also contributed significantly to the English grammar task that tapped into productive morphological knowledge. L1 skills thus lay the foundation for FL proficiency both directly and indirectly. Further research has to be done to understand an effect of task: both the effect of the task used to measure the grammatical abilities in L1 and the tasks used to measure grammatical learning in the FL. In all, our results underline the link between lexical and grammatical representations, as proposed by the framework of usage based grammar: our grammatical measures were related to vocabulary in both the L1 and the FL, and FL morphological knowledge seems mediated by L1 vocabulary knowledge and L1 grammar skills. This means that vocabulary skills are closely linked to grammar skills in an FL and this is already the case for beginning learners in an educational setting.

## Footnotes

1 L2 is used for those studies where English is (one of) the official language(s) of the larger speech community, whereas FL is used for those studies where English is not an official language of the larger speech community. Hence, when discussing the present study English is referred to as an FL, as it is not part of the official languages of the Netherlands.

2 English as a foreign language is a compulsory subject from grade 5 (ages 10–11 years) onwards in the Netherlands (Toorenburg & van Oostdam, 2002). However, primary schools may decide to offer English language lessons from an earlier grade onwards. Of the current 152 participants who were in grade 4, 113 pupils received English lessons at school and 39 pupils did not. No significant differences were found on the relevant tasks between the participants who had received English lessons at schools and those who had not, see Appendix. Therefore, we cannot conclude that there is a difference between these participants. This may seem surprising; however, all children in the Netherlands are exposed to English by means of television, music and computer games. In addition, the English lessons that the children before grade 5 receive have a typical duration of 15 min per week, in which they often learn to sing songs.

3 Originally there were 60 items in sets 7–11 of the PPVT-III-NL and all of these items were administered. However, since one item (*groente* ‘vegetable’) was also used in the English version of the PPVT, it was excluded from the analysis.

4 The frequency of the words in the L1 sentence assembly task was checked in the SUBTLEX-NL corpus (Keuleers & Brysbaert, 2010). All of the words had a logarithmic frequency of > 1.25, meaning that there were at least 17 counts on a total of 44 million tokens. Moreover, all of the words – apart from *bergbeklimmer* (‘mountaineer’) and *versturen* (‘to send’) – appeared in the Hazenberg and Hulstijn (1996) list of 23,550 basic Dutch words. *Berg* (‘mountain’), *beklimmen* (‘to climb’) and *sturen* (‘to send’) were listed amongst the 23,550 basic Dutch words.

5 Although the PPVT is developed for measuring native speakers’ vocabulary knowledge and may therefore be somewhat inappropriate for learners of English with a limited proficiency (see Goriot et al., 2018b); at the time of testing this was the best available vocabulary measure that had comparable English and Dutch counterparts.

6 Originally there were 84 items in sets 1–7 of the PPVT 4th edition and all of these items were administered. However, since one item (*vegetable*) was also used in the Dutch version of the PPVT and since other items were Dutch cognates, they were excluded from the analysis. Cognates were removed, because we wanted to ascertain the effect of Dutch on English and cognates would inflate this effect. Baird, Palacios, & Kibler (2016) indicate that “[c]ognates [are] words that are semantically and phonologically or orthographically similar in two languages”. The authors of the present paper checked the sets of the PPVT that were used and based their decision on the phonological representation of the items. Only when the authors agreed unanimously, the item counted as a cognate.
